# Testicular tuberculosis

**DOI:** 10.1590/0037-8682-0221-2023

**Published:** 2023-07-24

**Authors:** Juliana Garcia Alves da Trindade, Ana Caroline Siquara-de-Sousa, Diogo Goulart Corrêa

**Affiliations:** 1 Universidade Federal Fluminense, Departamento de Radiologia, Niterói, RJ, Brasil. Universidade Federal Fluminense Departamento de Radiologia Niterói RJ Brasil; 2 Universidade Federal Fluminense, Departamento de Patologia, Niterói, RJ, Brasil. Universidade Federal Fluminense Departamento de Patologia Niterói RJ Brasil

**FIGURE 1:**
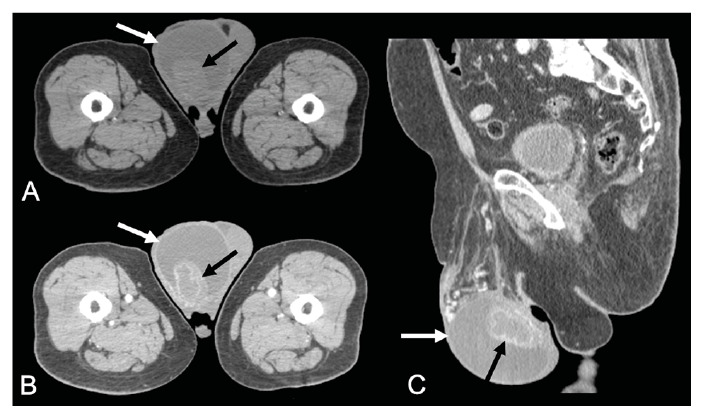
Pelvic computed tomography in the axial **(A and B)** and sagittal **(C)** planes, before **(A)** and after **(B and C)** intravenous contrast-injection showing a large hydrocele in the right scrotum (white arrows), associated with irregular right testicle and epididymis, with peripheral contrast-enhancement, and a central necrotic portion (black arrows).

**FIGURE 2:**
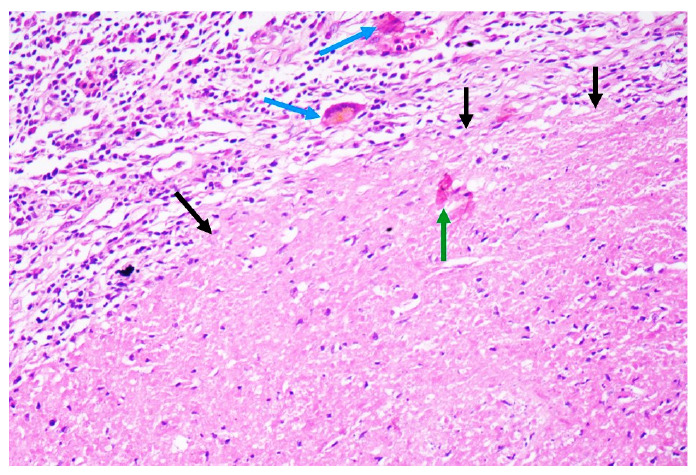
Histopathology of a hematoxylin and eosin-stained section of the testis (original magnification ×200) showing chronic granulomatous inflammation with extensive central caseous necrosis (black arrows) and multinucleated Langhans giant cells of the (blue arrows), and dystrophic calcification (green arrow), suggestive of tuberculosis. Wadefite, periodic acid-Schiff, and Grocott stains were negative for fungi and *Mycobacterium leprae*.

A 72-year-old man presented with painful enlargement of the scrotum and weight loss. Physical examination revealed that the right testicle and epididymis were hardened and tender. Hematology and chest radiography results were normal. Pelvic computed tomography (CT) revealed bilateral hydrocele and an irregular right testicle and epididymis with peripheral contrast enhancement and central necrotic hypodense portions (Figure 1). Radical orchiectomy was performed because of a suspected testicular tumor. Histopathology of the testis revealed chronic granulomatous inflammation with central caseous necrosis and multinucleated giant (foreign body and Langhans) cells, suggestive of tuberculosis (Figure 2). The patient was treated with rifampicin, isoniazid, pyrazinamide, and ethambutol.

Genitourinary tuberculosis is the second most common form of extrapulmonary tuberculosis after lymph node involvement; however, tuberculous orchiepididymitis is rare. Ultrasound may show focal or diffuse areas of hypoechogenicity in the testicle and epididymis, with increased surrounding vascularity on color Doppler imaging, and a hydrocele. CT usually shows irregular testicular masses with heterogeneous or peripheral contrast enhancement, and a hydrocele, with or without calcification^1^. Magnetic resonance imaging generally shows heterogeneous intrascrotal signal intensity with multiloculated and peripherally gadolinium-enhancing collections^2^. However, these findings are nonspecific and tuberculous orchiepididymitis cannot be differentiated from testicular neoplasms and non-specific orchiepididymitis based on imaging alone^1^^,^^2^. Typical findings of pulmonary tuberculosis may provide a clue to the diagnosis, but these are not always present. Histopathological analysis remains the gold standard for diagnosis^1^. Cytopathological analysis of samples obtained by ultrasound-guided fine-needle aspiration may avoid unnecessary surgery^3^.
