# Erratum

**DOI:** 10.1590/0037-8682-0221B-2023

**Published:** 2025-09-29

**Testicular tuberculosis**

In the document: Trindade JGAD, Siquara-de-Sousa AC, Corrêa DG. **Testicular tuberculosis. Rev Soc Bras Med Trop. 2023 Jul 24;56:e02212023.** doi: 10.1590/0037-8682-0221-2023.

**Page 1/1 (Figure 2 - Caption)**

**Figure 2** caption, where it reads Wadefite, **is correct** as Wade. 

**Figure 2** caption, where it reads *Mycobacterium leprae*, **is correct** as *Mycobacterium tuberculosis*. 

